# Linezolid to treat urinary tract infections caused by vancomycin-resistant *Enterococcus*

**DOI:** 10.1177/2050312120970743

**Published:** 2020-11-04

**Authors:** Benjamin Alan Pontefract, Suzette Amy Rovelsky, Karl Joseph Madaras-Kelly

**Affiliations:** 1 College of Pharmacy, Ferris State University, Big Rapids, MI, USA; 2 Boise Veterans Affairs Medical Center, Boise, ID, USA; 3 College of Pharmacy, Idaho State University, Meridian, ID, USA

**Keywords:** Infectious diseases, linezolid, urinary tract infections, antibiotic resistance, *Enterococcus*

## Abstract

**Background:**

Vancomycin-resistant *Enterococcus* can cause urinary tract infection. Linezolid possesses antimicrobial activity against vancomycin-resistant *Enterococcus* but has limited urinary excretion. Minimal data demonstrate efficacy of linezolid for treatment of urinary tract infections.

**Objective:**

The main aim of this study is to compare post-treatment outcomes of linezolid to other antibiotics with vancomycin-resistant *Enterococcus* activity in the treatment of urinary tract infection caused by vancomycin-resistant *Enterococcus*.

**Methods:**

A retrospective cohort of inpatients within Veterans Health Administration facilities with urinary tract infection caused by vancomycin-resistant *Enterococcus* was created. Patients with vancomycin-resistant *Enterococcus* isolated from urine cultures and chart documentation meeting criteria for urinary tract infection were identified. Demographics, comorbidity, treatments, and post-treatment outcomes were extracted from the electronic health record. Outcomes were compared between patients treated with linezolid and alternative antibiotics possessing vancomycin-resistant *Enterococcus* activity 14 days after treatment completion. Logistic regression adjusted for covariates associated with each outcome.

**Results:**

Of 4,683 patients with a positive vancomycin-resistant *Enterococcus* culture, 624 (13%) met criteria for chart review, and 92 (15%) had documentation of urinary tract infection symptoms and treatment. The primary reason for exclusion was asymptomatic bacteriuria (64%). Patients had high Charlson Comorbidity Scores (mean = 8.7; standard deviation (SD) = 3.3), and 70% were located on general medical/surgical wards on the day of culture collection. Linezolid was prescribed in 54 (59%) cases. No difference between linezolid and comparator antibiotics were observed in re-initiation of antibiotics for vancomycin-resistant *Enterococcus* urinary tract infection (9% and 5% respectively (p = 0.56), (adjusted odds ratio (OR) = 1.90; 95% confidence interval (CI) = 0.34–10.63)), recurrent positive vancomycin-resistant *Enterococcus* culture (4% and 11%, respectively (p = 0.23), (adjusted OR = 0.36; 95% CI = 0.05–2.31)), or mortality (7% and 3%, respectively (p = 0.39) (adjusted OR = 2.96; 95% CI = 0.37–41.39)).

**Conclusion:**

Most patients with vancomycin-resistant *Enterococcus* identified on urine culture were asymptomatic. Linezolid appears effective as comparator antibiotics for the treatment of mild vancomycin-resistant *Enterococcus* urinary tract infection.

## Introduction

*Enterococcus* is a frequently identified organism in urine cultures obtained in the hospital setting and can cause healthcare-associated urinary tract infections (UTIs).^1^ *Enterococcus* spp. recovered in the hospital setting are often resistant to vancomycin; these organisms are often referred to as vancomycin-resistant *Enterococcus* (VRE).^1^ Patients with enterococcal bacteriuria are often asymptomatic, and no treatment is indicated. However, enterococcal bacteriuria can also result in UTIs, particularly in patients with malignancies. These infections can progress to bacteremia or endocarditis if not appropriately managed.^2–4^ Treatment options for UTIs caused by vancomycin-resistant *Enterococcus* species include inexpensive and effective options such as aminopenicillins, tetracyclines, nitrofurantoin, and fosfomycin, but these options are not always feasible treatment options. *Enterococcus faecium* is often resistant to vancomycin as well as aminopenicillins and tetracyclines. Furthermore, nitrofurantoin and fosfomycin are not appropriate to use in the case of pyelonephritis due to poor penetration into kidney tissue. Antibiotics that possess consistent coverage against multi-drug-resistant VRE are generally expensive and mostly available as intravenous (IV) formulations (e.g. daptomycin and quinupristin/dalfopristin).

Linezolid is an antibiotic that possesses antimicrobial activity against VRE and is available as a generic oral formulation; however, references vary on whether linezolid should be used for this indication due to a lack of clinical use studies.^5–9^ One reason often cited against using linezolid for UTIs is that the linezolid package insert states that only 30% of each dose is excreted unchanged in the urine.^5^ To evaluate this statement, a study by Wagenlehner et al.^10^ evaluated 12 patients and demonstrated that linezolid is excreted unchanged in the urine in concentrations of 82–192 mg/L in the 12 h following a 600-mg oral dose in patients with normal kidney function. The Clinical and Laboratory Standards Institute’s (CLSI) breakpoints for *Enterococcus* species for linezolid are <2 μg/mL for susceptible strains, 4 μg/mL for intermediate susceptibility strains, and >8 μg/mL in resistant strains.^11^ When the CLSI breakpoints are compared to the results of the Wagenlehner study, it suggests linezolid enters urine in concentrations that are adequate to treat UTIs, but clinical use trials evaluating this hypothesis are limited.

Two clinical studies have evaluated the use of linezolid to treat UTI caused by VRE. The first study by Birmingham et al.^12^ was an observational trial that evaluated the use of linezolid to treat multidrug-resistant, gram-positive infections through a compassionate use program. This study included 34 patients with UTIs caused by VRE. Of these, 93% achieved clinical cure and 95% achieved microbiological cure. The second study by Rayner et al.^13^ was also an observational trial that evaluated the use of linezolid in the same compassionate use program. This study included 14 patients with UTIs caused by VRE. Of these, 100% achieved clinical cure and 83% achieved microbiological eradication. These data further suggest linezolid efficacy in the treatment of UTIs, but both of these trials only evaluated 48 patients between them, and neither included a comparator group. The purpose of this study was to compare post-treatment outcomes of linezolid with other antibiotics that possess activity against VRE for the treatment of VRE UTI.

## Methods

A national retrospective cohort of patients hospitalized within Veterans Health Administration (VHA) facilities who were diagnosed with a UTI with VRE isolated from urine culture between January 2012 and December 2018 was created. Patients with cultures obtained within 12 h preceding inpatient admission or collected during hospitalization that subsequently grew >10^3^ colony forming units (CFU)/mL of VRE were identified through the VA corporate data warehouse (CDW).^14^ The location of urine culture collection was used to define treatment location. Next, patients with >10^3^ VRE isolated from urine culture with antibiotics prescribed <3 days after culture collection were identified. Patients who did not receive an antibiotic with intrinsic activity against VRE were excluded. Antibiotics with intrinsic activity against VRE were identified using product package inserts, or CLSI standards for antimicrobial susceptibility testing.^5,15–23^ Based on susceptibility to ampicillin, susceptibility to amoxicillin and piperacillin/tazobactam was assumed.^24^ Susceptibility of patient-specific VRE was cross-referenced with administered antibiotics, and patients who received antibiotics to which their specific VRE was resistant were excluded unless another antibiotic with activity against their specific VRE was also given. For remaining patients, demographic, vital sign, laboratory value, and comorbidity data were extracted from the CDW. Finally, chart review of these patients’ electronic medical record (e.g. VA Clinical Patient Record System) was conducted to identify documented urinary tract signs and symptoms, urinary catheter use, antibiotic treatments, and post-treatment outcomes of interest. Chart review also confirmed if patients met documentation of clinical criteria for UTI.

Criteria for UTI diagnosis required that patients had a urine culture with a clinically sufficient quantity of VRE isolated and documentation of symptoms consistent with a UTI. Patients must have met one of the following UTI definitions: (1) >10^5^ CFU/mL of VRE growth with no invasive catheter use <48 h before culture, and at least one symptom of UTI on the day of culture or (2) >10^3^ CFU/mL of VRE growth, use of intermittent or indwelling urethral catheter within 48 h of urine collection, and at least one symptom of UTI or catheter-associated UTI on the same day as urine collection. These definitions were adapted from practice guidelines.^25,26^ Symptoms of UTI were defined by chart documentation of >1 of the following: increased urinary frequency, urinary urgency, dysuria, fever (defined as a single temperature greater than 38.3°C (101°F) or a temperature greater than 38.0°C (100.4°F) sustained over 1 h), flank pain, or costovertebral angle tenderness.^25,26^ Symptoms of catheter-associated UTI was defined as rigors, acute hematuria, or pelvic discomfort.^26,27^ Patients not meeting documented criteria for a UTI were excluded.

Patients were also excluded if they were a direct transfer to or from a non-VHA acute care hospital, administered an antibiotic with activity against VRE within 3 days prior to index culture collection, had >2 organisms reported on index urine culture, received <3 days of VRE active antibiotics, had withdrawal of care or death after index urine culture collection but before 3 days of antibiotic administration with VRE activity, or had therapy changed from a non-linezolid antibiotic to linezolid within 3 days of therapy initiation.

Definitive antibiotic therapy was classified as the first antibiotic administered with activity against the isolated VRE initiated <3 days after urine culture collection. Patients were stratified based on receipt of definitive antibiotic therapy into those that received linezolid or comparator antibiotic, which was defined as any non-linezolid antibiotic with activity against the identified VRE isolate. Comparator therapies without specific antibiotic susceptibilities reported were considered susceptible based on product inserts.^15,18,19,21^

Length of definitive therapy was reported, and post-treatment endpoints that occurred within 14 days of the end of definitive antibiotic treatment were evaluated. Endpoints included VRE persistence in urine culture and re-initiation of antibiotics with VRE activity. VRE persistence in urine culture was defined as a new urine culture that had growth of VRE. Patients who did not have a urine culture obtained during this endpoint evaluation time period were considered to be negative for VRE persistence. Re-initiation of antibiotics with VRE activity was defined as receipt of at least one dose of antibiotic with intrinsic activity against VRE in this endpoint evaluation time period regardless of whether the patient had a repeat urine culture that was positive for VRE or not. All-cause mortality was measured as death that occurred between day 4 of definitive antibiotic treatment and 14 days after the end of definitive antibiotic treatment. This timeframe was chosen to capture mortality influenced by the definitive antibiotic used; deaths on days 1 through 3 of definitive therapy are more likely to be due to factors other than the specific antibiotic given. The end date of definitive antibiotic treatment was based on the combination of IV or oral therapy administered as an inpatient plus the dispensed days’ supply of oral outpatient therapy.^28^

Demographic characteristics were compared with chi-squared test and contingency tables for categorical data and Student’s T-test for continuous data. The margin of significance for post hoc tests was defined as a two-tailed p value less than 0.05. Logistic regression was utilized to identify patient-level non-treatment covariates associated with each study endpoint (R version, 3.5.1). Covariates associated with each endpoint of interest at p < 0.1 level were used to develop a model for each endpoint based on minimizing Akaike Information Criterion. The definitive antibiotic group (linezolid or comparator) was added to the model to obtain adjusted estimates of treatment effect. Results are presented as unadjusted and adjusted odds ratios (ORs) with 95% confidence intervals (CIs).

This research complied with all federal guidelines and Department of Veterans Affairs policies relative to human subjects research (e.g. approved by the Institutional Review Board).

## Results

**Figure 1.**
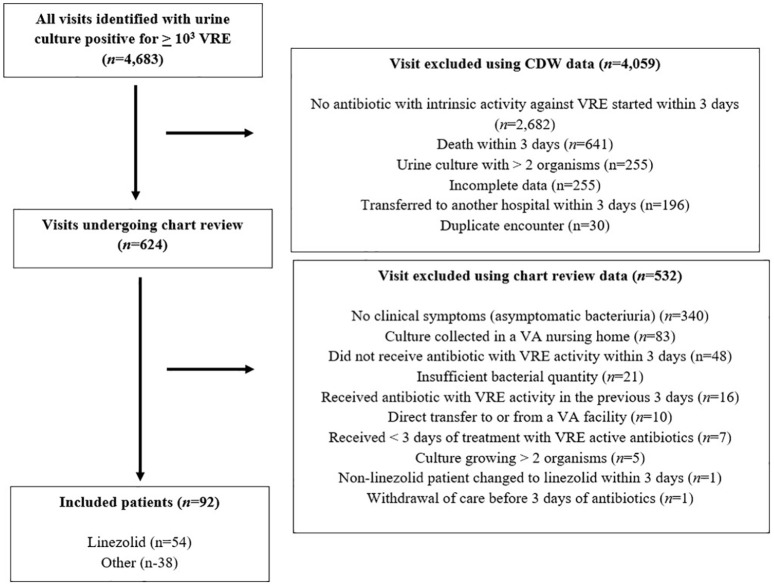
Flow diagram for identification of patients with UTI caused by VRE cohort. CDW: corporate data warehouse; UTI: urinary tract infection; VA: veterans affairs; VRE: vancomycin-resistant *Enterococcus*. Incomplete data refers to patients who did not have adequate data needed for identification within the electronic medical record. All timeframes refer to the date when the urine culture that grew VRE was collected.

A total of 4683 urine cultures with VRE isolated were identified. Of these, 4059 were excluded using data obtained directly within the CDW (Figure 1). The most common reason for exclusion during CDW data extraction was that the patient did not receive an antibiotic with intrinsic activity against VRE within 3 days of culture (66%, (2682/4059)) followed by death within 3 days of culture (16%, (641/4059)). The remaining 624 patients had their electronic medical record reviewed. Of these, 92 (15%) met all inclusion criteria and no exclusion criteria. The most common reason for exclusion during chart review was asymptomatic bacteriuria (ASB) (64%, (340/532)) followed by culture collection in non-inpatient setting (16%, (83/532)). Notably, 39% (131/340) of patients with ASB received antibiotics with VRE activity after culture collection.

Patients were mostly male and had a urinary catheter in place at the time of culture collection (Table 1). Patients generally had substantial comorbidity, had growth of *E. faecium* in urine culture and were managed outside of an intensive care unit (ICU). There was no significant difference between treatment groups in demographic characteristics or presenting symptoms of UTI.

**Table 1.** Patient demographics.^a^

	Total cohort (N = 92)	Linezolid (N = 54)	Comparators (N = 38)	p value
Age (years)	69 ± 12.5	68 ± 12.7	70 ± 12.2	0.45
Male gender	84 (91)	48 (89)	36 (95)	0.46
Catheter present	77 (84)	45 (83)	32 (84)	1.00
Urinary pathology^b^	58 (63)	35 (65)	23 (61)	0.83
Urinary procedure in previous 30 days^c^	10 (11)	8 (15)	2 (5)	0.19
Malignancy	43 (47)	25 (46)	18 (47)	1.00
Charlson Comorbidity Index	8.7 ± 3.3	8.9 ± 3.1	8.3 ± 3.5	0.39
*Enterococcus* species				
*E. faecium*	58 (63)	39 (72)	19 (50)	0.237
*E. faecalis*	25 (27)	7 (13)	18 (47)	0.002
Other	2 (2)	1 (2)	1 (3)	1.000
Unspecified	7 (8)	7 (13)	0 (0)	0.168
Location^d^				
ICU	21 (30)	16 (35)	5 (20)	0.302
Non-ICU	50 (70)	30 (65)	20 (80)	

ICU: intensive care unit.

a Data presented as n (%) or mean ± SD.

b Urinary pathology included the presence of urinary stent, current urolithiasis, benign prostatic hyperplasia, urinary flow obstruction, prostate cancer, bladder cancer, or nephrology tubes.

c Urinary procedures included urinary stent placement, urolithiasis management, nephrostomy tube placement, and transurethral resection of bladder tumor.

d Location was not specified for 21 patients. These data were available for 71 patients in the total cohort. This included 46 patients in the linezolid group and 25 patients in the comparator group.

The most common definitive antibiotic administered was linezolid (59%, (54/92)). Comparator antibiotics included penicillins (13% (12/92)), nitrofurantoin (12% (11/92)), daptomycin (8%, (7/92)), tetracyclines (7%, (6/92)), fosfomycin (1%, (1/92)), and quinupristin/dalfopristin (1%, (1/92)). Change in definitive antibiotic therapy (after >3 days) occurred in 6%, (3/54) of patients in the linezolid group, and 13%, (5/38) of patients in the comparator group (p = 0.15). In the linezolid group, changes were due to drug interaction with concurrent psychiatric medications (2%, (1/54)), the development of thrombocytopenia (2%, (1/54)), and de-escalation to ampicillin (2%, (1/54)). In the comparator group, 8% (3/38) of changes were transitions from IV to oral medication, 3% (1/38) continued febrile illness on nitrofurantoin, and 3% (1/38) de-escalation from tigecycline to linezolid.

Length of definitive treatment was similar between groups. The average (mean (±standard deviation)) duration was (11.5 (7.0)) days: (10.7 (4.8)) days in the linezolid group, and (12.1 (8.2)) days in the comparator antibiotic group (p = 0.31). Post-treatment outcomes within 14 days of the end of definitive antibiotic treatment for patients treated with linezolid and comparator antibiotics were similar. A total of 3% (3/92) of patients had a new urine culture with VRE identified and the presence of new urinary symptoms: 1 (2%) in the linezolid group and 2 (5%) of the comparator group (p = 0.57). Positive VRE urine culture alone was identified in 7% (6/92) of patients: 2 (4%) in the linezolid group and 4 (11%) in the comparator group (p = 0.23). Re-initiation of antibiotics with VRE activity occurred in 8% (7/92) of patients: 5 (9%) in the linezolid group and 2 (5%) in the comparator group (p = 0.56). Death occurred in 5% (5/92) of patients: 4 (7%) in the linezolid group and 1 (3%) in the comparator group (p = 0.39). Significant covariates associated with each outcome were limited. No significant differences in unadjusted or adjusted odds of any post-treatment outcome for linezolid relative to comparator antibiotics were observed (Table 2).

**Table 2.** Odds ratio (95% confidence intervals) for 14-day clinical outcomes comparing linezolid with comparator antibiotics.

	Unadjusted outcomes	Unadjusted p values	Adjusted outcomes	Adjusted p values
Positive VRE urine culture^a^	0.32 (0.06–1.85)	0.22	0.36 (0.05–2.31)	0.28
Re-initiation of antibiotics with VRE activity^b^	1.82 (0.33–9.97)	0.69	1.90 (0.34–10.63)	0.46
Mortality^c^	2.96 (0.32–27.59)	0.40	2.96 (0.37–41.39)	0.34

UTI: urinary tract infection; VRE: vancomycin-resistant *Enterococcus*.

Included mortality from day 4 of definitive treatment to 14 days after the end of definitive treatment.

a Positive VRE urine culture covariates (univariate OR (95% CI)): 30-day history of urological procedure (9.25 (1.67–51.28)).

b VRE UTI Retreatment Covariates (univariate OR (95% CI)): 30-day history of urological procedure (7.32 (1.41–37.95)).

c Mortality covariates (univariate OR (95% CI)): length of definitive treatment (0.68 (0.48–0.96)).

## Discussion

The results of this retrospective analysis identified that linezolid is frequently prescribed for treatment of UTI caused by VRE. We observed that in patients with documented UTI symptoms who were managed outside of the ICU, linezolid demonstrated similar efficacy to comparator antibiotics. Another notable observation is that over one-third (131/340 (39%)) of patients with VRE isolated but without documented symptoms of UTI (e.g. ASB) were treated with antibiotics.

A strength of this analysis was the use of the VA’s CDW to identify a national cohort of patients with a urine culture positive for VRE. While study criteria were applied within the CDW; chart-level evaluation was also utilized to confirm documentation of UTI symptoms, treatments, and post-treatment outcomes that could not be readily captured through database extraction. Another strength includes the use of definitions for diagnoses that were congruent with national guidelines in order to minimize the number of ASB cases included in the cohort.

There are also several study limitations. First, we excluded all patients who had withdrawal of care or death after urine culture collection but before 3 days of definitive antibiotic treatment was administered. This allowed assessment of the association between administration of definitive antibiotic treatment and study endpoints. However, it also contributed to a cohort that primarily received management in non-ICU settings. It is possible that administration of antibiotic therapy within the initial 3-day timeframe impacted early death or withdrawal of care; therefore, these data should not be extrapolated to patients with severe UTI caused by VRE. An additional limitation is the relatively small evaluative population. Despite the national scale of the evaluation, the narrow diagnostic and treatment criteria excluded the majority of patients with VRE recovered from urine cultures. This smaller sample size, along with relatively few endpoint events, limited power to detect type II error for difference in outcomes. Still, this is the largest study to date evaluating the efficacy of linezolid for VRE UTIs. Also, some patients received several doses of antibiotics with activity against the cultured VRE isolate that were not their definitive antibiotic. In total, 3/54 (6%) of patients in the linezolid group received at least one dose of a non-linezolid antibiotic, and 2/38 (5%) of patients in the comparator group received at least one dose of linezolid. Finally, treatment location data was not specified for 23% of our patients, but a univariate analysis of treatment location on each of the endpoints did not show significant impact.

Our findings are similar to those previously reported. First, VRE recovered from urine, particularly in catheterized patients, is more often associated with ASB than true infection. Wong et al.^4^ reported that 59% of patients with urine cultures with VRE isolated in a tertiary care facility had either ASB or colonization. This is similar to our findings that in which identified that 57% of patients with VRE in the urine were not treated with a VRE-active antibiotic, which suggests a high rate of ASB. Our study also corroborated that VRE infections are more commonly reported in patients with a high degree of comorbidity.^29–32^ Our post-treatment outcome results are similar to the two open-label, non-comparative, non-randomized studies previously discussed, which showed a clinical cure rate of 93% and 100%. Our study evaluated twice the number of patients as these previous studies to further support the hypothesis that linezolid can effectively treat UTI caused by VRE.^12,13^

## Conclusion

We observed the majority of urine cultures identified with VRE were submitted from patients with ASB. In a cohort of patients with symptomatic VRE UTI, associated catheter use, and low acuity of illness, treatment with linezolid resulted in similar outcomes as comparator antibiotics. The findings suggest that linezolid is as effective as other VRE active antibiotics in the treatment of mild UTI caused by VRE.
